# Effects of exercise training on frailty, cardiorespiratory fitness, and lower limb function in non-dialysis chronic kidney disease: a systematic review and meta-analysis

**DOI:** 10.3389/fmed.2026.1868774

**Published:** 2026-07-20

**Authors:** Sujie Yang, Fan Zhang, Xueling Li, Wen Zhu, Qiuye Chen, Yifei Zhong, Xianwen Zhang

**Affiliations:** Longhua Hospital, Shanghai University of Traditional Chinese Medicine, Shanghai, China

**Keywords:** cardiorespiratory fitness, chronic kidney disease, exercise training, fraity, lower limb function, meta-analysis

## Abstract

**Introduction:**

Physical frailty, impaired cardiorespiratory fitness (CRF), and diminished lower limb function are prevalent in non-dialysis chronic kidney disease (NDD-CKD) and strongly associated with adverse clinical outcomes. This systematic review and meta-analysis aimed to evaluate the efficacy of exercise training interventions on the core dimensions of the frailty phenotype, CRF, and lower limb function in the NDD-CKD population.

**Methods:**

Seven electronic databases were systematically searched from inception to March 1, 2026, for randomized controlled trials (RCTs) comparing structured exercise interventions with usual care or sham exercise in adult NDD-CKD patients. Primary outcomes included the five core frailty phenotypes (absolute body weight, handgrip strength, walking speed, physical activity, and fatigue), CRF, and lower limb function. Data were synthesized using a random-effects model, reporting standardized mean differences (SMDs) with 95% confidence intervals (CIs). This study is registered with PROSPERO (CRD420261363651).

**Results:**

Eighteen RCTs encompassing 938 participants were included. Compared with the control group, exercise interventions significantly improved specific frailty dimensions, notably increasing walking speed (SMD = 0.36, 95% CI 0.03–0.69; *P* = 0.03) and alleviating fatigue (SMD = 0.34, 95% CI 0.04–0.64; *P* = 0.03). Furthermore, exercise significantly enhanced overall CRF (SMD = 0.46, 95% CI 0.29–0.63; *P* < 0.001) and lower limb function (SMD = 0.55, 95% CI 0.32–0.79; *P* < 0.001). Outcomes for absolute body weight, handgrip strength, and general physical activity levels did not reach statistical significance.

**Conclusion:**

Exercise training is an effective and safe intervention method that can alleviate specific frailty characteristics (walking speed and fatigue), and enhance the cardiovascular and lower extremity functions of NDD-CKD patients. It is advocated to promote exercise as an early “pre-rehabilitation” strategy to enhance the physical functional reserve of patients before dialysis.

**Systematic review registration:**

https://www.crd.york.ac.uk/prospero/, identifier CRD420261363651.

## Introduction

Chronic kidney disease (CKD) is a major global public health challenge, affecting approximately 10–12% of the population worldwide (1). It leads to progressive renal failure and is strongly associated with cardiovascular mortality and end-stage kidney disease (ESKD) (2). Alongside these “hard” outcomes, CKD is increasingly characterized by diminished physiological reserve and severe functional impairment that adversely affect daily life and prognosis (3).

Cardiorespiratory fitness (CRF) is substantially impaired across the CKD continuum (4). Meta-analytic evidence shows that VO_2_peak is significantly lower in CKD patients compared with healthy controls (SMD -1.40), with a progressive decline as kidney function worsens (4, 5). Concurrently, frailty, a multidimensional syndrome characterized by decreased physiological reserve and heightened vulnerability to stressors, is highly prevalent in the CKD population (3). Given the strong links between functional limitation, frailty, cardiovascular risk, and mortality, exercise has been increasingly advocated as a core nonpharmacologic strategy to preserve health and functional independence across disease stages (6). Although early rehabilitation literature primarily emphasized dialysis populations, an expanding body of randomized controlled trials (RCTs) suggests that structured exercise is also highly beneficial in non-dialysis-dependent CKD (NDD-CKD) (7, 8). Recent meta-analyses show that exercise interventions significantly improve VO_2_peak, 6-min walk distance (6MWD), and Timed Up and Go (TUG) test performance in NDD-CKD patients, metrics closely aligned with lower-limb function and frailty phenotypes (8, 9).

Despite guideline endorsement and a growing evidence base, critical research gaps remain for NDD-CKD patients, particularly concerning outcomes that map directly onto frailty and its functional components (10). Therefore, a focused quantitative synthesis of exercise effects in the NDD-CKD population is warranted. Although existing meta-analyses have examined exercise in pre-dialysis CKD (8, 11), none has simultaneously quantified the five individual components of the Fried physical frailty phenotype, CRF, and lower-limb function within a single synthesis restricted exclusively to the NDD-CKD population. The most recent and comprehensive review by Traise et al. (8) addressed exercise capacity and walking distance broadly but did not decompose frailty into its constituent phenotypic dimensions. Zhang et al. (12) conducted a meta-analysis on nutritional supplementation combined with exercise interventions in CKD patients, evaluating each independent component of the Fried phenotype separately but focused primarily on dialysis populations. The present systematic review and meta-analysis addresses these gaps by: (1) providing a disaggregated, component-level analysis of the frailty phenotype; (2) incorporating an updated search inclusive of Chinese-language databases; and (3) offering subgroup analyses by exercise modality and assessment tool to support more actionable clinical guidance. This systematic review and meta-analysis aims to evaluate the effects of exercise interventions on frailty status, CRF, and performance-based lower-limb function specifically in adults with NDD-CKD.

## Materials and methods

The protocol of this systematic review and meta-analysis was prospectively registered at PROSPERO (CRD420261363651) and written following the Preferred Reporting Items for Systematic Reviews and Meta-Analyses (PRISMA) reporting guidelines (13). This study was exempt from ethics review because it is a pooled analysis of published data.

### Search strategy

We systematically searched seven electronic databases, including PubMed, Embase, Web of Science, Cochrane Central Register of Controlled Trials (CENTRAL), China National Knowledge Infrastructure (CNKI), Wanfang Data, and SinoMed, from inception to March 1, 2026.We adopted a comprehensive retrieval strategy that combines medical subject heading (MeSH) terms and synonyms to retrieve eligible studies fully. Moreover, we manually investigated a relevant reference of systematic reviews (8, 11) to search for additional potential studies. The search strategies are available in the attachment. Any disagreement about retrieving eligible studies was solved by consulting a third author.

### Study selection

After the initial search, two authors independently screened article titles and abstracts to identify potentially relevant studies. Two authors then independently screened and reviewed full-text articles based on inclusion and exclusion criteria. Disagreements were resolved by a third author.

### Eligibility criteria

Table 1 reports the study’s “Population, Intervention, Comparator, Outcomes, and Study design” inclusion criteria. A study was excluded if it met at least one of the following criteria: (i) patients affected by acute kidney injury or requiring maintenance dialysis; (ii) conference abstracts, case reports, study protocols, or reviews; (iii) written in languages other than English or Chinese; (iv) when studies with duplicate populations or overlapping data were identified, we chose to include the study with the most comprehensive data or the largest sample size; (v) studies focusing exclusively on dietary/nutritional therapy without a structured exercise intervention, or lacking available data for the targeted outcomes.

**TABLE 1:** Study inclusion criteria, defined by the PICOS.

PICOS component	Description
Patient	Adults (aged ≥ 18 years) diagnosed with non-dialysis chronic kidney disease (CKD stages 1–5, not requiring renal replacement therapy), irrespective of the specific estimated glomerular filtration rate (eGFR) threshold used for inclusion, provided that CKD was defined according to established guidelines (e.g., KDIGO).
Intervention	Structured exercise training programs (e.g., aerobic training, resistance training, or combined exercise).
Comparasion	Usual care, standard medical management, wait-list, or non-exercise/sham control groups (e.g., simple stretching, health education).
Outcomes	Reporting at least one of the following domains: 1. Frailty phenotypes: including core dimensions such as absolute body weight (strictly excluding Body Mass Index [BMI]), upper limb static strength (e.g., handgrip strength, walking speed, physical activity levels, and fatigue/vitality scores). 2. Cardiorespiratory fitness: e.g., peak oxygen uptake (VO_2_peak), 6MWD. 3. Lower limb function: e.g., sit-to-stand (STS) tests, SPPB, lower limb muscle strength.
Study design	RCTs

### Quality assessment

Two independent authors assessed the risk of bias for each included RCT according to the Cochrane Collaboration’s risk of bias tool 2(RoB2) as having a low risk of bias, some concerns, or a high risk of bias: bias arising from the randomization process, bias due to deviations from intended interventions, bias due to missing outcome data, bias in measuring the outcome and bias in the selection of the reported result (14). The Excel macro tool provided on the RoB2 official website^1^ was used to generate the risk of bias summary table. Any disagreement about the methodological quality assessment was resolved by consulting a third author.

### Statistical analysis

Two independent investigators systematically extracted data from the eligible studies using a standardized electronic spreadsheet. The extracted baseline characteristics included the first author, publication year, sample size, demographic profiles (age and gender), baseline CKD stage, and detailed protocols for both the intervention and control groups. For the targeted clinical outcomes, specifically, the five core dimensions of the frailty phenotype (absolute body weight, handgrip strength, walking speed, physical activity, and fatigue/vitality), cardiorespiratory fitness (e.g.,VO_2_peak, 6MWD), and lower limb function, post-intervention means and standard deviations (SDs) were extracted. In trials reporting outcomes across multiple follow-up time points, data from the final assessment were preferentially utilized to capture the complete intervention effect.

To rigorously prevent unit-of-analysis errors and double-counting bias in multi-arm trials (15, 16), we did not divide the sample size of the shared control group. Instead, in accordance with the Cochrane Handbook for Systematic Reviews of Interventions regarding the handling of multiple intervention groups, we strategically selected the single intervention arm that most closely aligned with our predefined inclusion criteria to compare directly against the control group, deliberately excluding the other less relevant arms from that specific meta-analysis. Furthermore, an a priori “hierarchy of evidence” was applied when studies reported multiple assessment tools for a single physiological domain. For instance, if lower extremity function was assessed using both STS tests and isokinetic knee extension strength, two senior authors collaboratively evaluated the clinical relevance and extracted the single most appropriate objective measure to maintain the statistical independence of the pooled effect sizes.

All quantitative syntheses for continuous outcomes (frailty phenotypes, cardiorespiratory fitness, and lower limb function) were conducted using the standardized mean difference (SMD) with 95% confidence intervals (CIs). To ensure consistency and comparability across the included trials, data harmonization was meticulously performed. When continuous outcomes were reported in varying units, they were converted to a standardized metric. Crucially, to align the directionality of clinical benefit, the extracted mean values of functional tests where lower scores indicate better performance or pathological “decline” (e.g., fatigue scores, walking speed decline) were mathematically inverted (i.e., multiplied by -1) prior to the pooled analysis. This standardization ensured that a positive SMD consistently denoted a favorable clinical improvement in the exercise group. When a standard error of the mean (SEM) was reported, it was converted to a standard deviation (SD) using the formula: SD = SEM ×√n (where n is the number of subjects). For data reported as medians and quartiles, conversions to sample means and SDs were performed utilizing the methods of Luo et al. (17) and Wan et al. (18). Any discrepancies arising during the data extraction and harmonization process were resolved through consensus or by consulting a third senior reviewer.

**TABLE 2:** Characteristic of the included studies.

Study (reference)	Mean age at baseline (years)	Gender (M/F)	CKD stage	Intervention (setting, type, dose)	Control	Outcome
Chen et al. (16)	Int: 75.6 ± 5.2 Con: 72.8 ± 5.1	Int: 27/5 Con: 22/5	Stages 2–4	CT + flexibility, 30–45 min, 3–5 days/wk, 3 months	Usual care	Frailty: Fatigue score, Grip strength decline, Walking speed decline, Physical activity decline Lower Limb: 30s-STS(n)
Castaneda et al. (22)	Int: 65.0 ± 9.0 Con: 64.0 ± 13.0	Int: 8/6 Con: 9/3	Moderate impairment	RT (80% 1RM), 45 min, 3 days/wk, 12 weeks	Sham exercise	Frailty: Body weight (kg) Lower limb: Knee extension (kg)
Watson et al. (23)	Int: 63 [57–65] Con: 66 [63–72]	Int: 11/9 Con: 14/4	Stages 3b-4	RT (70% 1RM), 3 days/wk, 8 weeks	Usual care	Cardiorespiratory: ISWT (m), ESWT (min) Lower limb: Isometric strength (Nm)
Uchiyama et al. (24)	Int: 72 (69–79) Con: 76 (69–78)	Int: 17/6 Con: 16/7	Stage 4	Home-based CT (AT 3 days/wk; RT 2 days/wk), 6 months	Usual care	Frailty: Handgrip strength (kg) Cardiorespiratory: ISWT (m) Lower limb: Quadriceps strength (kg)
Aoike et al. (15)	Int: 56.0 ± 8.3 Con: 54.3 ± 8.7	Int: 8/4 Con: 10/5	Stages 3–4	Home-based AT, 30–50 min, 3 days/wk, 24 weeks	Usual care	Cardiorespiratory: VO2peak (mL/kg/min), 6MWD (m) Lower limb: 30s-STS (n)
Begue et al. (25)	Int: 62.6 ± 10.8 Con: 67.2 ± 8.2	Int: 12/11 Con: 2/7	Stages 3–5	Home-based CT (HIIT + RT), 30–40 min, 3 days/wk, 12 weeks	Usual care	Frailty: Body weight (kg) Cardiorespiratory: 6MWD (m), VO2peak (mL/kg/min)
Nixon et al. (19)	Int: 77.0 ± 8.3 Con: 78.8 ± 7.0	Int: 9/6 Con: 7/7	Stages 3b-5	Home-based CT ( + balance), 30–45 min, 3 days/wk, 12 weeks	Usual care	Frailty: Walking speed (m/s) Lower limb: SPPB
Tang et al. (26)	Int:46.3 ± 15.6 Con:43.9 ± 12.4	Int:28/14 Con:23/19	Stages 1–3	Home-based AT, 20–30 min, ≥ 3 days/wk, 12 weeks	Usual care	Cardiorespiratory: 6MWD (m) Lower Limb: STS10 (s)
Tabata et al. (27)	Int: 74.7 ± 6.3 Con: 74.6 ± 5.4	Int: 9/6 Con: 9/5	Stages 3–5	Home-based CT, 6 months	Usual care	Frailty: Handgrip strength (kg), Walking speed (m/s) Cardiorespiratory: 6MWD (m) Lower limb: SPPB
Rossi et al. (21)	Int: 68.0 ± 12.0 Con: 69.0 ± 12.0	Int: 23/36 Con: 33/15	Stages 3–4	Center-based CT ( + flexibility), 2 days/wk, 24 sessions	Usual care	Cardiorespiratory: 6MWT (ft) Lower limb: STST (% age pred)
Weiner et al. (28)	Int: 67.9 ± 7.7 Con: 68.1 ± 8.8	Int: 34/15 Con: 40/10	Stages 3b-4	Center-based CT, 3 days/wk, 6 months	Health education	Cardiorespiratory: VO2peak (mL/kg/min), 6MWD (feet)
Kirkman et al. (20)	Int: 54.0 ± 13.0 Con: 55.0 ± 12.0	Int: 11/7 Con: 10/8	Stage 3	AT (70–85% HRmax), 45 min, 3 days/wk, 12 weeks	Usual care	Frailty: Weight (kg), speed (4 m, s), Grip (kg) Cardiorespiratory: VO2peak (mL/kg) Lower limb: 30s-STS (n)
Howden et al. (32)	Int: 58.3 ± 9.7 Con: 59.8 ± 10.2	Int: 23/13 Con: 22/14	Stages 3–4	CT (8-wk center + 10-mo home-based), 12 months	Usual care	Frailty:Weight (kg), Grip (kg), TUG (s) Cardiorespiratory: 6MWD (m)
de Araújo et al. (29)	Int:57.9 ± 2.7 Con:58.1 ± 5.2	Int:9/7 Con:9/6	Stage 2	Home-based RT, 22 weeks	Usual care	Frailty: Grip (kgf) Cardiorespiratory: 6MWT (m) Lower limb: TUG (s)
Barcellos et al. (33)	Int: 61.2 ± 11.9 Con: 60.1 ± 12.3	Int: 47/29 Con: 41/33	Stages 2–4	CT, 60 min, 3 days/wk, 16 weeks	Usual care	Frailty: weight (kg) Lower limb: 30s-STS (n)
Leehey et al. (30)	Int:65.4 ± 8.7 Con:66.6 ± 7.5	Int:18/0 Con:18/0	Stages 2–4	Center-based CT, 12 weeks	Diet management	Cardiorespiratory: 6MWD (m) Lower limb: TUG (s)
Ikizler et al. (31)	Int: 54.0 ± 10.0 Con: 53.0 ± 12.0	Int: 17/13 Con: 15/16	Stages 3–4	Center-based AT, 3 days/wk, 4 months	Usual care	Frailty: Weight (kg) Cardiorespiratory: VO2peak (mL/kg) Lower limb: SPPB (score)
Hiraki et al. (34)	Int: 69.0 ± 6.8 Con: 67.8 ± 6.9	Int: 14/0 Con: 14/0	Stages 3–4	Home-based CT, 12 months	Usual care	Frailty: Grip (kgf) Lower limb: Knee Ext (kgf/kg)

Data are presented as mean ± SD or median [interquartile range]. Weiner et al. (28) included an additional 6-month maintenance phase. Int, intervention group; Con, control group; M, male; F, female; AT, aerobic training; RT, resistance training; CT, combined training (aerobic + resistance); HIIT, high-intensity interval training; 1RM, one-repetition maximum; HRmax, maximal heart rate.

Given the anticipated clinical and methodological variations inherent in diverse exercise interventions, a random-effects model utilizing the restricted maximum likelihood (REML) estimation method was applied for all quantitative meta-analyses. Statistical heterogeneity across the included trials was evaluated using Cochrane’s Q test and the *I*^2^ statistic, with *I*^2^ values of 25, 50, and 75% representing low, moderate, and substantial heterogeneity, respectively. To explore potential sources of heterogeneity, predefined subgroup analyses were explicitly conducted based on intervention modalities (i.e., aerobic exercise, resistance training, and combined training) and the specific assessment tools utilized for lower limb function. Furthermore, random-effects meta-regression analyses were performed for continuous covariates, such as mean patient age and intervention duration, to further elucidate the variance in pooled effect sizes. All statistical analyses and graphical representations (including forest plots, funnel plots, and meta-regression bubble plots) were executed using the meta package in R software. A two-sided *P* < 0.05 was considered statistically significant for all tests.

## Results

### Identification of eligible studies

A total of 418 relevant records were initially identified from seven electronic databases. After removing 85 duplicate records, 333 records were screened by title and abstract, of which 283 were excluded as they were not relevant to the research topic. Fifty reports were sought for full-text retrieval, but 1 report could not be retrieved. Therefore, 49 full-text articles were rigorously assessed for eligibility. During the full-text review stage, 31 articles were excluded for the following reasons: inappropriate intervention (*n* = 23), population not CKD (*n* = 2), duplicate data (*n* = 2), reviews (*n* = 2), conference paper (*n* = 1), and no extractable data available (*n* = 1). Finally,18 articles were included in both the systematic review and the quantitative meta-analysis (15, 16, 19–34). The entire review process, from the initial search to the final selection of studies, is illustrated in the PRISMA flowchart (Supplementary Figure S1). Table 2 shows the basic characteristics of the included studies. The bias assessment for each included RCT is shown in Supplementary Figure S2.

### Effects of exercise interventions on frailty phenotypes

**FIGURE 1:**
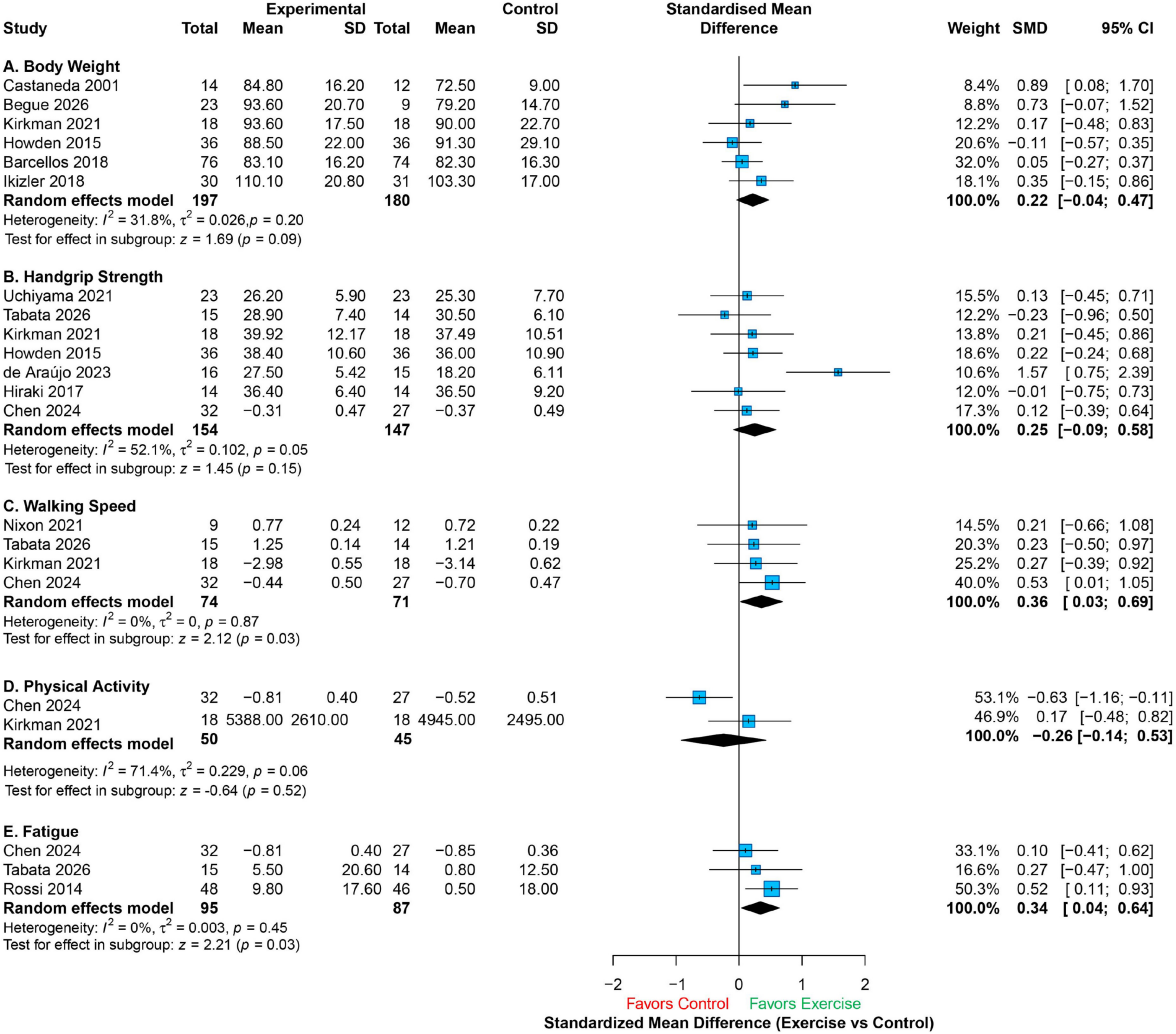
Forest plot evaluating the effects of exercise interventions on the five core dimensions of the frailty phenotype in patients with non-dialysis chronic kidney disease. **(A)** Body weight. **(B)** Handgrip strength. **(C)** Walking speed. **(D)** Physical activity. **(E)** Fatigue.

To comprehensively evaluate the effect of exercise on the frailty syndrome in non-dialysis patients with CKD, we conducted a quantitative comprehensive analysis of the five core dimensions of the classic Fried physical frailty phenotype (Figure 1).

Six studies (20, 22, 25, 31–33) (six trials, 377 participants) presented body weight outcomes. Meta-analysis showed that absolute body weight at the end of follow-up in the intervention group was, on average,0.22 SMD units higher than that of the control (95% CI -0.04 to 0.47). Although showing a trend toward improvement, there was no statistical difference (*P* = 0.09), with low-to-moderate statistical heterogeneity across the included trials (*I*^2^ = 31.8%, *P* = 0.20).

Seven articles (16, 20, 24, 27, 29, 32, 34) (seven trials, 301 participants) reported handgrip strength outcomes. Meta-analysis showed that handgrip strength was, on average, 0.25 SMD units higher in the intervention group than the control (95% CI -0.09 to 0.58), with no statistical difference (*P* = 0.15) and moderate statistical heterogeneity (*I*^2^ = 52.1%, *P* = 0.05).

Four articles (16, 19, 20, 27) (four trials, 145 participants) presented outcomes of walking speed. Meta-analysis showed that walking speed at the end of follow-up was significantly improved, being on average 0.36 SMD units higher in the intervention group than in the control (95% CI 0.03–0.69, *P* = 0.03). Furthermore, this subgroup demonstrated excellent statistical homogeneity (*I*^2^ = 0%, *P* = 0.87).

Two articles (16, 20) (three trials,95 participants) presented physical activity outcomes. Meta-analysis showed that physical activity levels in the exercise intervention group were comparable to those in the control group, with an average standardized mean difference (SMD) of -0.26 (95% CI: -1.04 to 0.53). The overall effect was not statistically significant (*P* = 0.52), and substantial statistical heterogeneity was observed between the included trials (*I*^2^ = 71.4%, *P* = 0.06).

Three articles (16, 21, 27) (three trials, 182 participants) reported fatigue or vitality outcomes. Meta-analysis showed that fatigue symptoms at the end of follow-up were significantly alleviated, with vitality scores being on average 0.34 SMD units higher in the intervention group than in the control (95% CI 0.04–0.64, *P* = 0.03). Furthermore, this subgroup demonstrated excellent statistical homogeneity (*I*^2^ = 0%, *P* = 0.45).

### Effects of exercise interventions on cardiorespiratory fitness

**FIGURE 2:**
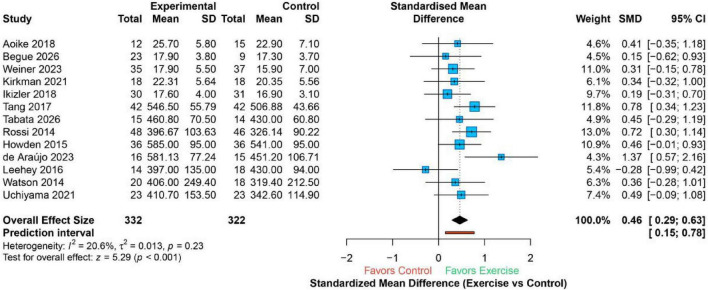
Forest plot evaluating the overall effect of exercise interventions on cardiorespiratory fitness in patients with non-dialysis chronic kidney disease.

Thirteen articles (15, 20, 21, 23–32) (13 trials, 654 participants) presented overall cardiorespiratory fitness outcomes. Meta-analysis showed that cardiorespiratory fitness at the end of follow-up in the intervention group was, on average, 0.46 SMD units higher than that of the control (95% CI 0.29–0.63), demonstrating a significant statistical difference (*P* < 0.001) with low statistical heterogeneity (*I*^2^ = 20.6%, *P* = 0.23) (Figure 2).

**FIGURE 3:**
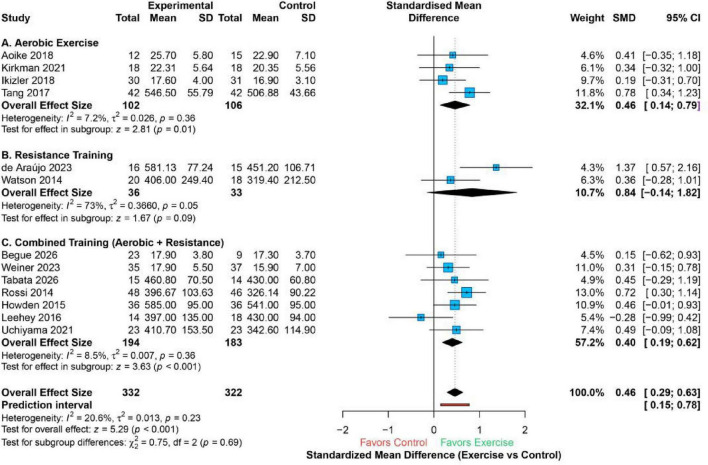
Subgroup analysis forest plot: effects of different exercise modalities on cardiorespiratory fitness. **(A)** Aerobic training; **(B)** resistance training; **(C)** combined training (aerobic + resistance).

Subgroup analyses further delineated the specific effects of different training modalities (Figure 3). Both aerobic exercise (15, 20, 26, 31) (four trials, 208 participants) and combined training (21, 24, 25, 27, 28, 30, 32) (seven trials, 377 participants) yielded highly homogeneous and significant benefits, improving cardiorespiratory fitness by 0.46 SMD units (95% CI 0.14–0.79, *P* = 0.01; *I*^2^ = 7.2%) and 0.40 SMD units (95% CI 0.19–0.62, *P* < 0.001; *I*^2^ = 8.5%), respectively. In contrast, while pure resistance training (23, 29) (two trials, 69 participants) showed an upward trend with a large effect size of 0.84 SMD units (95% CI -0.14 to 1.82), it did not reach statistical significance (*P* = 0.09) and exhibited substantial heterogeneity (*I*^2^ = 73.0%, *P* = 0.05).

### Effects of exercise interventions on lower limb function

**FIGURE 4:**
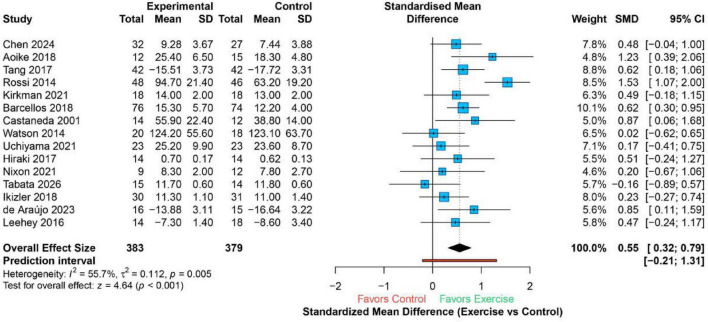
Forest plot evaluating the overall effect of exercise interventions on lower limb function in patients with non-dialysis chronic kidney disease.

Fifteen articles (15, 16, 19–24, 26, 27, 29, 31, 33–35) (15 trials, 762 participants) presented overall lower limb function outcomes. Meta-analysis showed that lower limb muscle strength and function at the end of follow-up in the intervention group were, on average, 0.55 SMD units higher than those of the control (95% CI 0.32–0.79), demonstrating a highly significant statistical difference (*P* < 0.001) with moderate statistical heterogeneity across the included trials (*I*^2^ = 55.7%, *P* = 0.005) (Figure 4).

**FIGURE 5:**
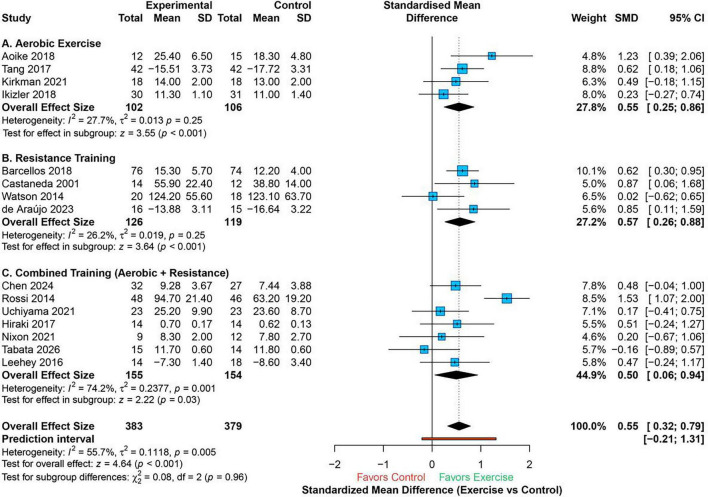
Subgroup analysis forest plot: effects of different exercise modalities on lower limb function. **(A)** Aerobic training; **(B)** resistance training; **(C)** combined training (aerobic + resistance).

Subgroup analyses further delineated the specific effects of different training modalities, revealing a universal responsiveness of the lower limb musculature to various forms of exercise (Figure 5). Both aerobic exercise (15, 20, 26, 31) (4 trials, 208 participants) and resistance training (22, 23, 29, 33) (4 trials, 245 participants) yielded significant and highly homogeneous benefits, improving lower limb function by 0.55 SMD units (95% CI 0.25–0.86, *P* < 0.001; *I*^2^ = 27.7%) and 0.57 SMD units (95% CI 0.26–0.88, *P* < 0.001; *I*^2^ = 26.2%), respectively. Similarly, combined training (16, 19, 21, 24, 27, 34, 35) (aerobic plus resistance;7 trials,309 participants) also significantly improved lower limb outcomes by 0.50 SMD units (95% CI 0.06–0.94, *P* = 0.03), although with higher observed statistical heterogeneity (*I*^2^ = 74.2%).

**FIGURE 6:**
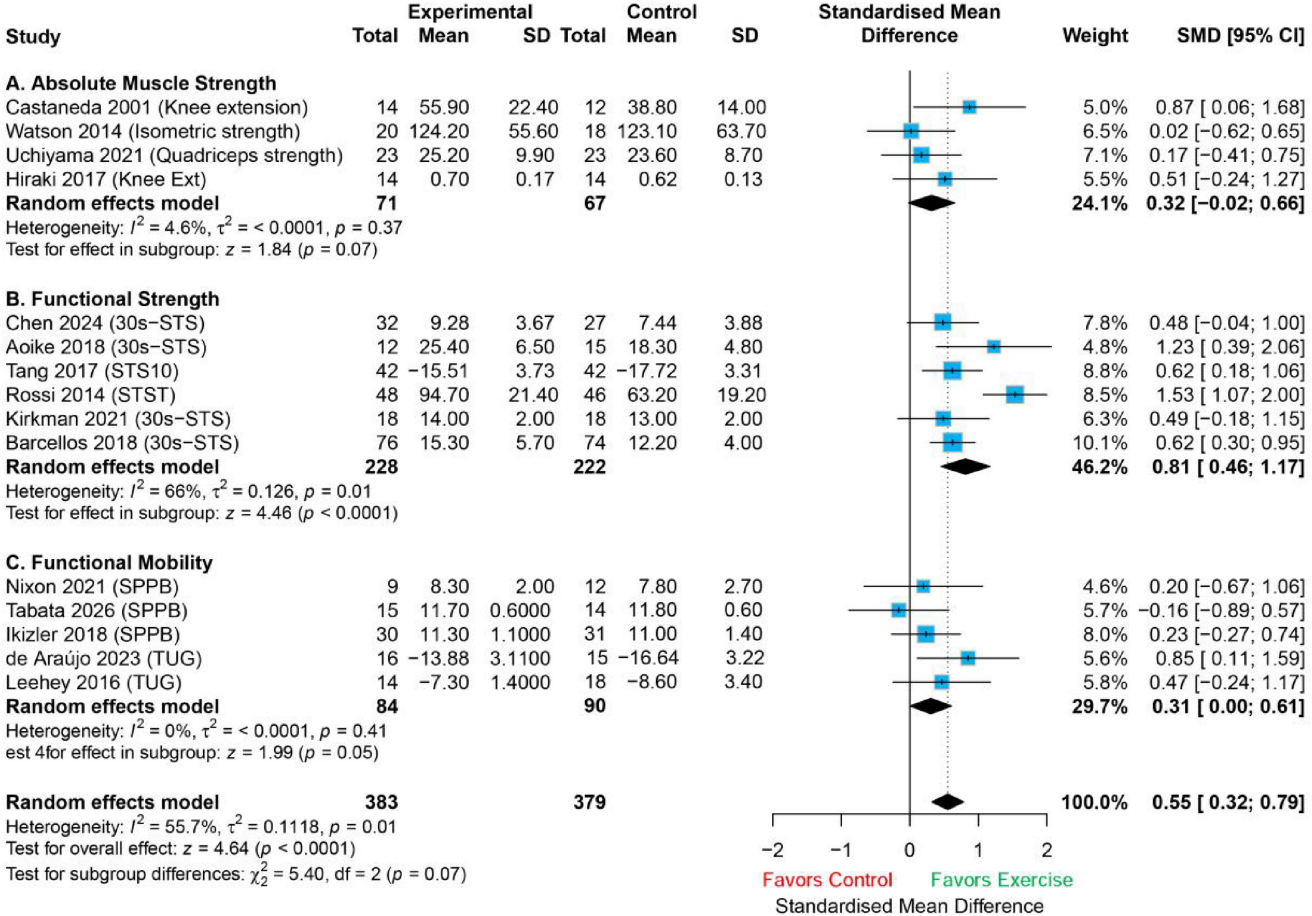
Subgroup analysis forest plot: effects of exercise interventions on lower limb function stratified by specific assessment outcomes. **(A)** Absolute muscle strength; **(B)** functional strength; **(C)** functional mobility.

Subgroup analyses further delineated the specific effects across different assessment outcomes, revealing a varied responsiveness of the lower limb to the interventions (Figure 6). Functional strength (15, 16, 20, 21, 26, 33) (6 trials, 450 participants) yielded significant benefits, improving lower limb outcomes by 0.81 SMD units (95% CI 0.46–1.17, *P* < 0.001), although with higher observed statistical heterogeneity (*I*^2^ = 66%). Similarly, functional mobility (19, 27, 29–31) (5 trials,174 participants) also significantly improved outcomes by 0.31 SMD units (95% CI 0.00–0.61, *P* = 0.05), demonstrating highly homogeneous results (*I*^2^ = 0%). In contrast, absolute muscle strength (22–24, 34) (4 trials, 138 participants) showed a positive trend but did not reach statistical significance, yielding an effect size of 0.32 SMD units (95% CI -0.02 to 0.66, *P* = 0.07; *I*^2^ = 4.6%).

### Sensitivity analysis

The corresponding “leave-one-out” results for each meta-analysis are shown in Supplementary Figures S3, S4.

### Meta-regression

Meta-regression analyses revealed that neither mean patient age nor intervention duration acted as significant moderators for the effect sizes of exercise interventions on cardiorespiratory fitness or lower limb function (all *P* > 0.05; detailed regression estimates are provided in Supplementary Table S1, and precision-weighted bubble plots are shown in Supplementary Figures S5, S6).

### Descriptive analysis

Among the 18 eligible RCTs, three studies conducted systematic evaluations of the composite frailty status in patients with NDD-CKD. Due to the substantial heterogeneity in assessment instruments—specifically the utilization of the Fried Frailty Phenotype (FFP) and the Edmonton Frail Scale (EFS)—and disparate data reporting formats, a descriptive synthesis was performed for this outcome.

The investigation by Chen et al. (16), involving 112 elderly patients with CKD stages 2–4, provides the primary quantitative evidence for frailty amelioration. Following the 12-week intervention, FFP scores significantly declined from baseline (*P* < 0.05), with the most pronounced improvements observed in the “Exercise + Enhanced External Counterpulsation (EECP)” cohort. Within the FFP sub-dimensions, the domains of “fatigue” and “low physical activity”—both central to the frailty syndrome—demonstrated the most significant clinical recovery.

In the EX-FRAIL CKD pilot trial, Nixon et al. (19) explored the efficacy of home-based multi-component exercise in frail or pre-frail patients. Although the sample size was modest (*N* = 29) and mean total scores were not reported, categorical transition data revealed a robust trend toward frailty reversal: 33.0% of participants in the exercise group transitioned to a more favorable status (e.g., from “frail” to “pre-frail”), compared to only 8.0% in the usual care group. This study further substantiated the feasibility of home-based exercise for managing the multidimensional nature of frailty using the EFS.

Additionally, the study by Aoike et al. (15) in overweight CKD patients observed that regular aerobic exercise not only enhanced cardiorespiratory endurance but also fostered a positive trajectory toward the reversal of the frailty phenotype.

In summary, despite the current scarcity of pooled quantitative data, consistent evidence from these trials indicates that structured exercise interventions—implemented in either supervised center-based or autonomous home-based settings—confer significant clinical benefits by attenuating frailty severity and facilitating the transition toward more robust physical states in the NDD-CKD population.

### Adverse events

All eighteen included studies provided information regarding the safety or occurrence of adverse events during the intervention periods. Specifically, Watson et al. (23) excluded five potential participants prior to randomization due to asymptomatic electrocardiogram abnormalities identified during safety screening. Nixon et al. (36) reported musculoskeletal pain (9 cases), one fall, nocturnal leg cramps, and postural dizziness in 15 exercising participants. Furthermore, while several studies recorded participant withdrawals due to non-related illnesses, injuries sustained outside the study, or the commencement of dialysis, no serious adverse events directly attributed to the exercise interventions were reported across the remaining trials. For instance, long-term interventions (up to 12 months) and high-intensity resistance training programs explicitly stated that no exercise-related injuries or cardiovascular accidents occurred (28). However, it should be noted that none of the included studies employed a consistent or standardized approach to proactively monitoring and reporting adverse events throughout the trial duration.

### Publication bias

Visual inspection of the funnel plots for the primary outcomes revealed a roughly symmetrical distribution of the effect sizes around the pooled estimates, suggesting an absence of apparent publication bias (Supplementary Figure S7). This visual observation was statistically corroborated by Egger’s linear regression test, which confirmed that no significant small-study effects or publication bias were present across the included trials (Egger’s test: *P* = 0.509 for cardiorespiratory fitness, and *P* = 0.492 for lower limb function).

## Discussion

### Summary of main results

This systematic review and meta-analysis included 18 trials encompassing 938 participants. All included trials compared structured exercise interventions to a control intervention (defined as usual care, lifestyle maintenance, or sham exercise). We found that exercise therapy yielded clinically important improvements in frailty characteristics within the NDD-CKD population compared with controls; from a statistical standpoint, it demonstrated robust significance particularly in mitigating fatigue, diminished walking speed, and physical inactivity. Synthesized evidence suggests that structured exercise compared with control substantially enhanced overall physical function by improving CRF and lower extremity neuromuscular capacity. Sensitivity analyses showed highly stable results across all meta-analyses. Furthermore, the meta-regression analysis indicated that the cardiorespiratory capacity and lower limb function were not related to the patient’s age or the duration of the intervention.

### Interpretation of the results

One of the advantages of this study lies in that it specifically examines the effects of exercise intervention on the frailty status, CRF and lower limb function of NDD-CKD. Because the therapeutic potential of exercise has been recognized throughout the course of CKD, but high-quality evidence regarding frailty and its specific components is more available from the dialysis population. For instance, an umbrella review by Zhang et al. (37) synthesized extensive meta-analytical evidence confirming that exercise interventions significantly improve exercise capacity and alleviate fatigue in patients with CKD. Furthermore, a recent meta-analysis by Zou et al. (38) reinforced these functional benefits in the end-stage setting, demonstrating that intradialytic exercise effectively mitigates overall frailty and enhances physical performance in maintenance hemodialysis patients.

Our systematic review also found that after undergoing exercise training, the fatigue and walking speed in the frailty dimension of patients with NDD-CKD were improved and showed statistical significance. It is important to acknowledge that only three of the 18 included studies assessed composite frailty status using a validated global instrument [i.e., the FFP (16) or the EFS (19)]. The remaining studies did not administer a summary frailty score; instead, they measured one or more of the five frailty phenotype components independently. This approach is conceptually defensible on several grounds. First, the Fried phenotype is operationally defined as a count of five discrete physical criteria—weight loss, exhaustion, low physical activity, slowness, and weakness—each of which is individually measurable and clinically meaningful (39). Second, a component-level approach is consistent with recommendations from exercise medicine, where domain-specific outcomes (e.g., gait speed, grip strength) are recognized as sensitive indicators of change that may precede shifts in composite frailty category (40). Third, performance-based measures such as gait speed and the TUG test have independently demonstrated predictive validity for mortality and disability in CKD populations. Nonetheless, we recognize that improvement in individual components does not necessarily equate to a change in overall frailty classification, and future trials should prioritize the concurrent use of validated composite frailty instruments alongside component-level assessments.

It is worth noting that the state of frailty is not irreversible. A longitudinal study found that during a 12-month follow-up, the improvement and progression of frailty occurred at a similar frequency, indicating its dynamic nature and the possibility of responding to intervention (41). This further supports the importance of early identification and intervention of frailty-related issues in CKD patients, with the aim of improving their quality of life or prognosis. Studies have shown that frailty can be identified early in CKD and progresses as renal function deteriorates (42). Therefore, interrupting this downward trajectory before patients reach ESKD is of paramount clinical importance. However, one study found that the minimum exercise intensity to inducemuscle hypertrophy or muscle fiber adaptation was as high as 80–95% 1RM (43), which is not permissible for most CKD patients, especially for those with a more dependent dialysis-dependent CKD population, whose exercise adherence is usually low due to cardiopulmonary dysfunction or physical limitations (44).

CRF holds extremely significant clinical importance in patients with CKD, and its significance is reflected in multiple aspects such as prognosis assessment, quality of life, and cardiovascular risk prediction. It is worth noting that although the cardiopulmonary fitness of CKD patients is lower than that of healthy controls, it is still better than that of heart failure patients. This suggests that despite functional impairment, CKD patients still retain relatively good exercise capacity (5). Our meta-analysis revealed the effectiveness of exercise intervention in enhancing the cardiopulmonary endurance of patients with NDD-CKD (SMD = 0.46, *P* < 0.001), and observed that both simple aerobic exercise and combined training could lead to stable and statistically significant improvements in cardiopulmonary function. This result can be corroborated by multiple studies (8, 11, 45). Aerobic exercise promotes continuous muscle contraction, enabling the heart and lungs to continuously supply oxygen to the muscles and remove metabolic waste, thereby effectively improving the cardiovascular and pulmonary functions (46). Resistance exercise is highly effective in enhancing muscle strength and quality, while the reduction in muscle mass is associated with a decline in mitochondrial respiratory function. It can also regulate inflammatory factors, thereby reducing renal fibrosis and inflammation, and possibly slowing the progression of CKD (47). In summary, exercise intervention improves the cardiovascular function, reduces inflammation, enhances muscle mass, and through multiple mechanisms, jointly improves the cardiopulmonary fitness of CKD patients. Based on these benefits of the mechanisms, we might be able to extend the treatment window for exercise to the pre-dialysis stage, in order to establish “cardio-pulmonary reserve.” The transition to ESKD and the initiation of dialysis often lead to a sharp decline in function and severe exercise intolerance, thereby compromising rehabilitation compliance. By leveraging the relatively preserved motor capacity during the NDD-CKD stage, early structured exercise improves the physiological baseline. When dialysis is inevitable, this accumulated reserve can play a certain buffering role.

The measurement values of lower limb function in CKD patients are significantly lower than those in the general population. A study showed that in patients with an average age of 61 and an average eGFR of 41 mL/min/1.73 m^2^, the performance of lower limb function was at least 30% lower than the predicted value of the general population (48), indicating that CKD itself has a significant negative impact on muscle function. CKD is a state of catabolism, accompanied by protein consumption, inflammation, fluid overload and malnutrition. These factors collectively lead to increased muscle breakdown, reduced muscle protein synthesis, and impaired muscle regeneration, ultimately resulting in muscle atrophy, decreased physical function and frailty (49). This condition is more severe compared to NDD-CKD individuals of the same age. A umbrella review indicates that muscle strength can be measured through grip strength and lower limb muscle strength, and in 10 meta-analyses,8 of them showed that exercise can improve muscle strength in patients with CKD (37). Our meta-analysis also confirmed that both aerobic exercise, resistance exercise, and combined exercise can effectively improve the lower limb function of patients with NDD-CKD. It is worth noting that in the subgroup analysis, the subgroups evaluated by TUG and SPPB showed extremely low heterogeneity (*I*^2^ = 0, *P* = 0.006). According to a systematic review, the SPPB, TUG, and STS-60 have strong evidence supporting their use in CKD (40). The SPPB score can predict mortality, frailty status and transplantation possibility and the TUG is recommended for assessing dynamic balance and activity ability in hemodialysis and CKD patients. Early identification of improvements in these indicators is likely to help reduce the score for physical weakness in patients with chronic kidney disease.

The improvement of CRF and lower limb function has profound clinical significance for patients with NDD-CKD, directly related to quality of life, disease progression and mortality risk. Physical frailty in CKD is fundamentally characterized by a depletion of physiological reserve, manifesting clinically as progressive exhaustion and impaired mobility (36). The concurrent improvement of CRF and lower limb function addresses multiple domains of this frailty phenotype. While enhanced CRF optimizes central cardiovascular capacity and mitigates systemic uremic fatigue, improvements in lower limb function provide the peripheral neuromuscular competence required for activities of daily living. By synergistically targeting these central and peripheral components, structured exercise effectively ameliorates the core “slowness” and “weakness” phenotypes, facilitating the transition from a frail state to increased functional independence (50). From a long-term prognostic perspective, the establishment of this cardiopulmonary and musculoskeletal “reserve” may significantly alter the clinical trajectory of patients with NDD-CKD. The progressive decline in renal function is frequently associated with a cycle of physical inactivity, accelerated muscle wasting, and increased cardiovascular morbidity (51). Proactively building physiological capacity during the pre-dialysis phase can potentially attenuate this downward spiral. Optimizing baseline functional status not only reduces the immediate risks of falls and incident disability but also enhances physiological tolerance for the hemodynamic and metabolic stresses associated with the transition to ESKD. Consequently, integrating exercise into early CKD management aligns with the contemporary therapeutic objective of prolonging functional healthspan and preserving patient autonomy, rather than solely delaying the initiation of renal replacement therapy (52).

## Limitations

Several limitations within this systematic review and meta-analysis should be acknowledged. First, although our analysis aggregated 18 trials encompassing 938 participants, the sample sizes of the individual studies were relatively small, and the intervention durations were predominantly short-to-medium term (ranging from 8 to 52 weeks). This precludes us from evaluating the long-term impact of exercise on hard clinical endpoints, such as progression to ESKD or all-cause mortality, which remains a prevalent challenge in nephrology rehabilitation research (53). Second, considerable clinical heterogeneity was present across the included trials and represents a substantive limitation. With regard to CKD stage, enrolled patients ranged from CKD stage 1 to stage 5 (pre-dialysis), and few studies stratified results by eGFR category; given that exercise capacity, muscle wasting, and frailty burden differ markedly across the CKD continuum, pooling participants across stages may obscure stage-specific treatment effects and limit the precision of clinical recommendations. With regard to exercise modality, included interventions encompassed aerobic training, progressive resistance training, and combined programs delivered in supervised center-based, home-based, and hybrid settings, with highly variable session frequencies (2–5 days/week), intensities (40–80% VO*2*peak or 50–80% 1RM), and session durations. With regard to intervention duration, programs ranged from 8 to 52 weeks, precluding firm conclusions about the minimum effective dose or the persistence of benefits beyond the intervention period. Although our predefined subgroup analyses by modality partially addressed exercise-type heterogeneity, the small number of trials within each subgroup limits the interpretability of these comparisons. As highlighted by international guidelines, the absence of standardized FITT prescriptions complicates the identification of an optimal exercise dose (54), and future trials should adopt consensus-based reporting frameworks to enable more meaningful cross-study comparisons. Third, while we observed significant improvements in specific frailty phenotypes, the assessment tools for frailty and physical function varied slightly among studies. Such methodological diversity in measuring multidimensional frailty may contribute to the statistical heterogeneity observed in certain subgroup analyses (36). Future large-scale, multicenter randomized controlled trials with extended follow-up periods are warranted to address these gaps and standardize exercise prescriptions.

## Conclusion

Our systematic review and meta-analysis indicate that for patients with NDD-CKD, exercise training can significantly improve frailty characteristics (such as fatigue, walking ability), cardiopulmonary fitness, and lower limb function, but other frailty characteristics (such as body weight, physical activity level, and grip strength) do not show improvement. Our study is a rigorous, up-to-date, and adequately powered meta-analysis specifically examining this pre-dialysis population. Considering the severe functional decline associated with the transition to end-stage renal disease and the dynamic nature of physical frailty, more effort should be taken to implement early structured exercise as an evidence-based “prehabilitation” strategy to establish physiological reserve in patients with NDD-CKD.

## Data Availability

The original contributions presented in this study are included in this article/Supplementary material, further inquiries can be directed to the corresponding authors.

## Footnotes

1
https://www.riskofbias.info/welcome
