# Supplementary figures and images for: Effects of exercise training on frailty, cardiorespiratory fitness, and lower limb function in non-dialysis chronic kidney disease: a systematic review and meta-analysis
Source: Front Med (Lausanne). 2026 Jul 20;13:1868774. doi: 10.3389/fmed.2026.1868774 (PMC13429782; doi:10.3389/fmed.2026.1868774)

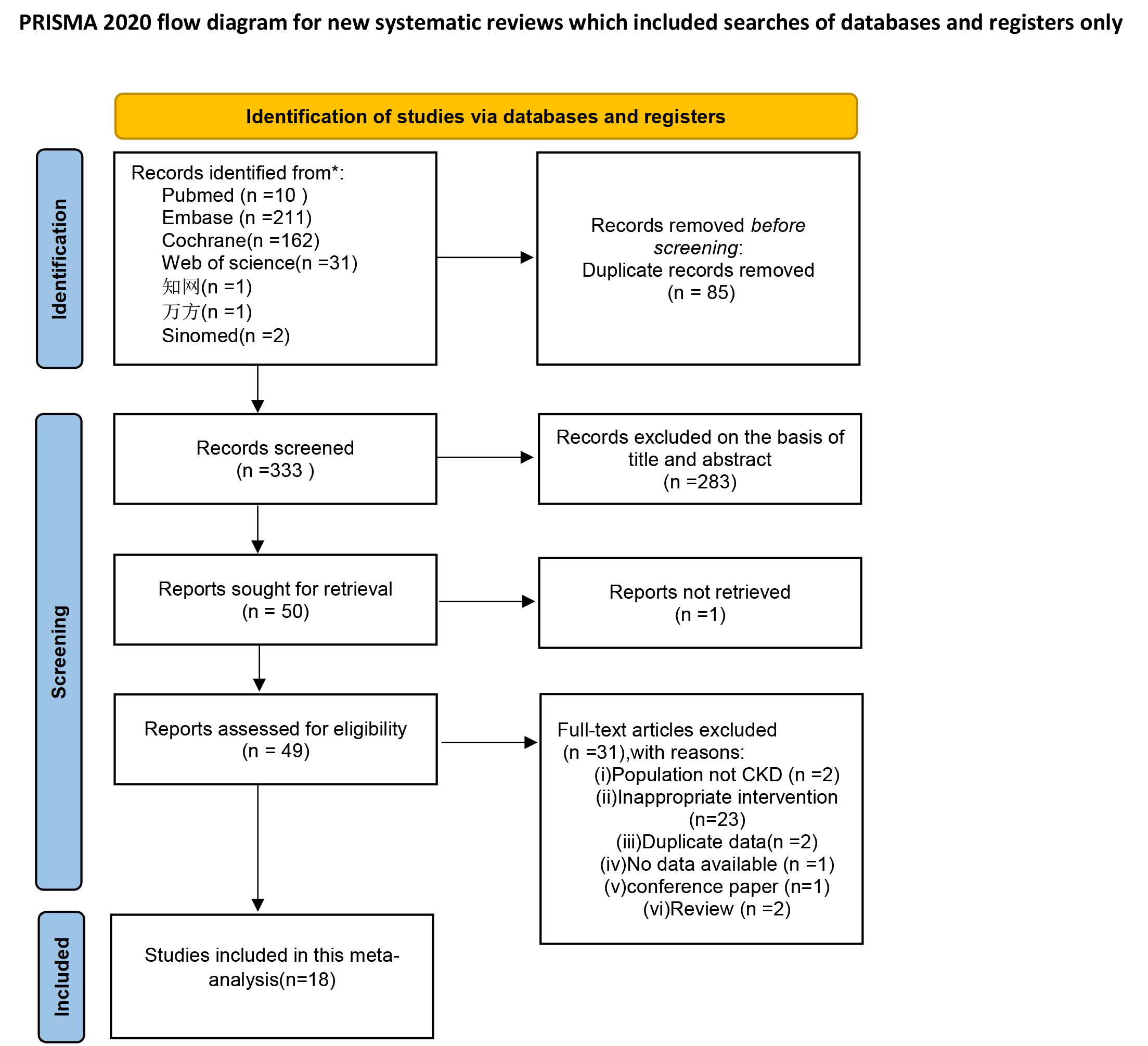

Supplement: Supplementary file 1 [file Presentation_1.zip › Supplementary Material/Figure A1. PRISMA flowchart.tif]

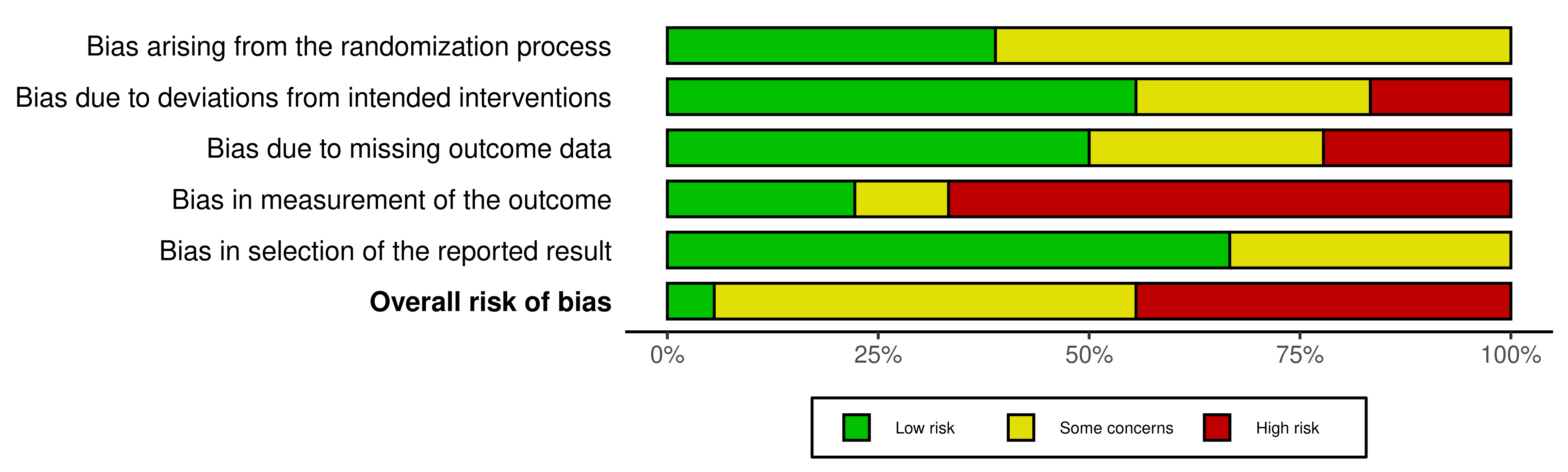

Supplement: Supplementary file 1 [file Presentation_1.zip › Supplementary Material/Figure A2. The bias assessment for each included RCT-ROB1.tiff]

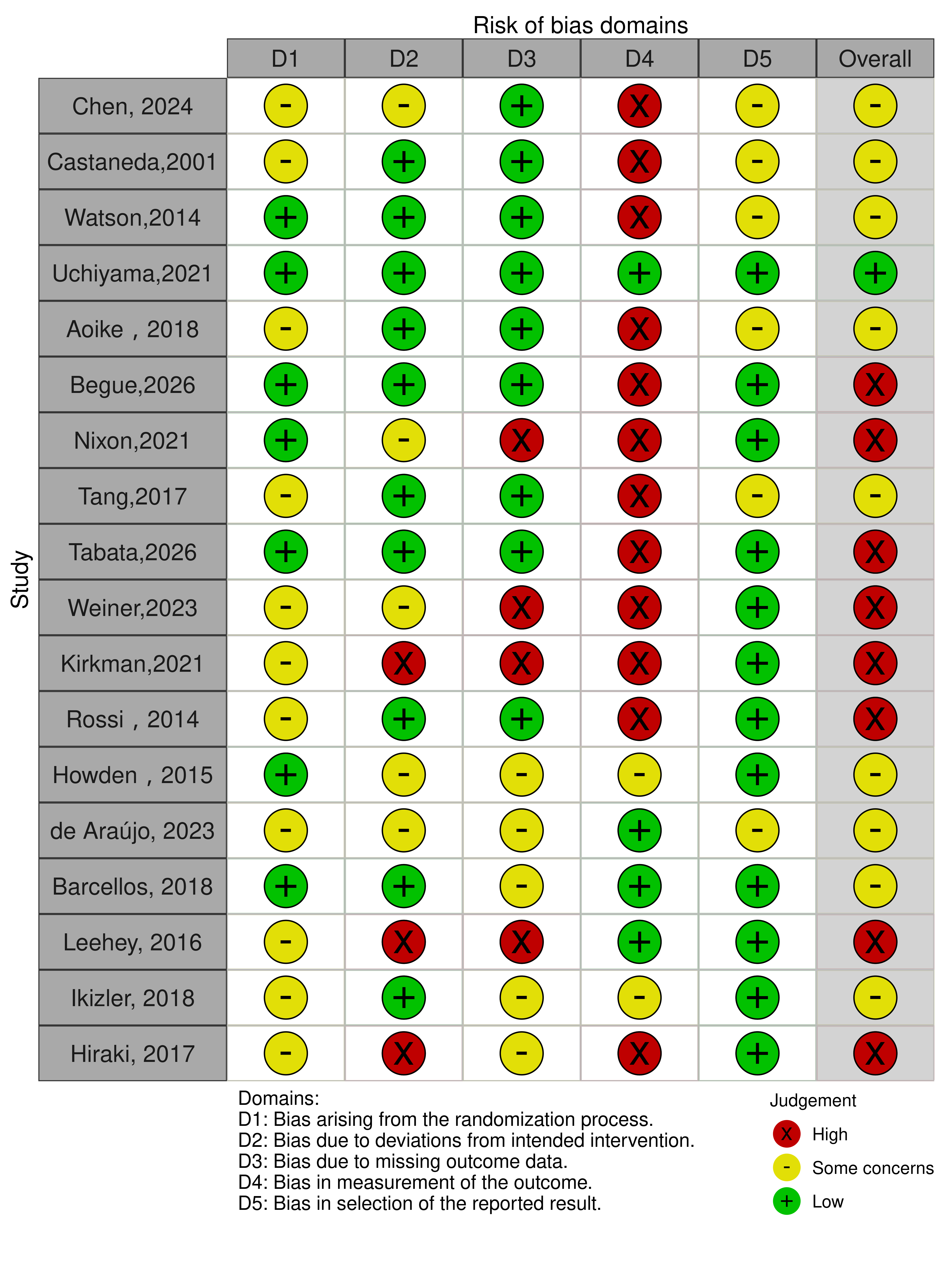

Supplement: Supplementary file 1 [file Presentation_1.zip › Supplementary Material/Figure A2. The bias assessment for each included RCT-ROB2.tiff]

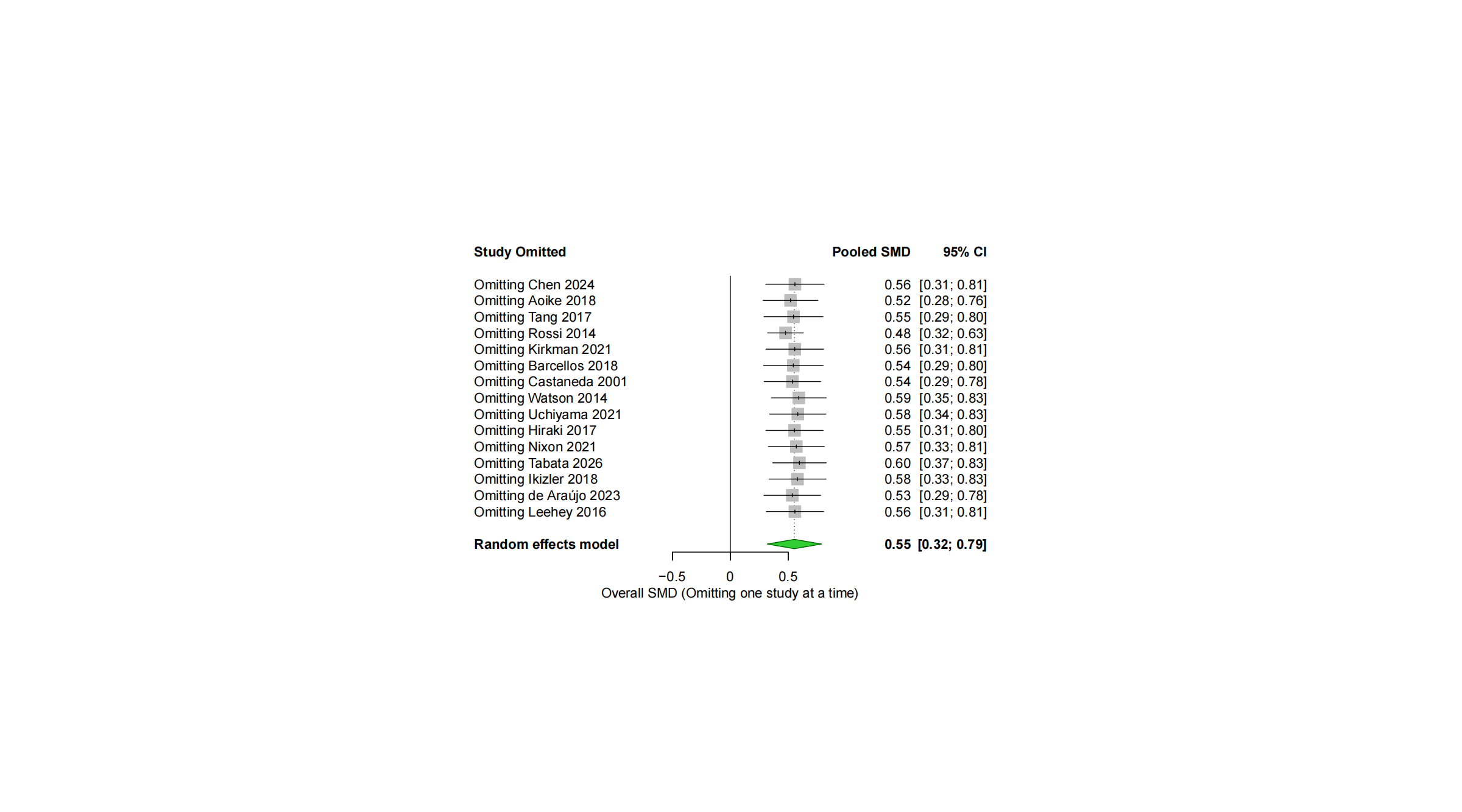

Supplement: Supplementary file 1 [file Presentation_1.zip › Supplementary Material/Figure A3. Leave-one-out sensitivity analysis forest plot for the effect of exercise interventions on cardiorespiratory fitness..tif]

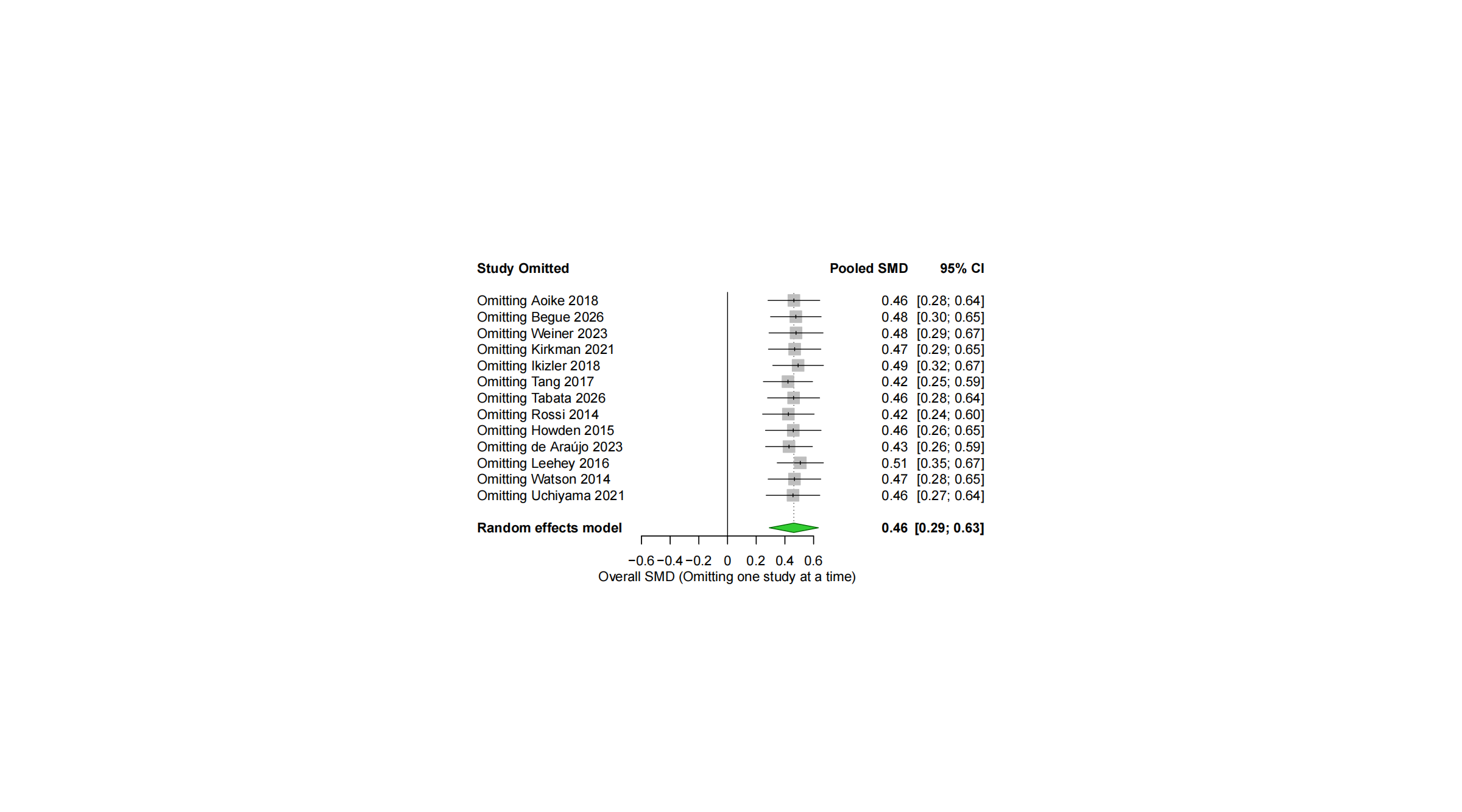

Supplement: Supplementary file 1 [file Presentation_1.zip › Supplementary Material/Figure A4. Leave-one-out sensitivity analysis forest plot for the effect of exercise interventions on lower limb function..tif]

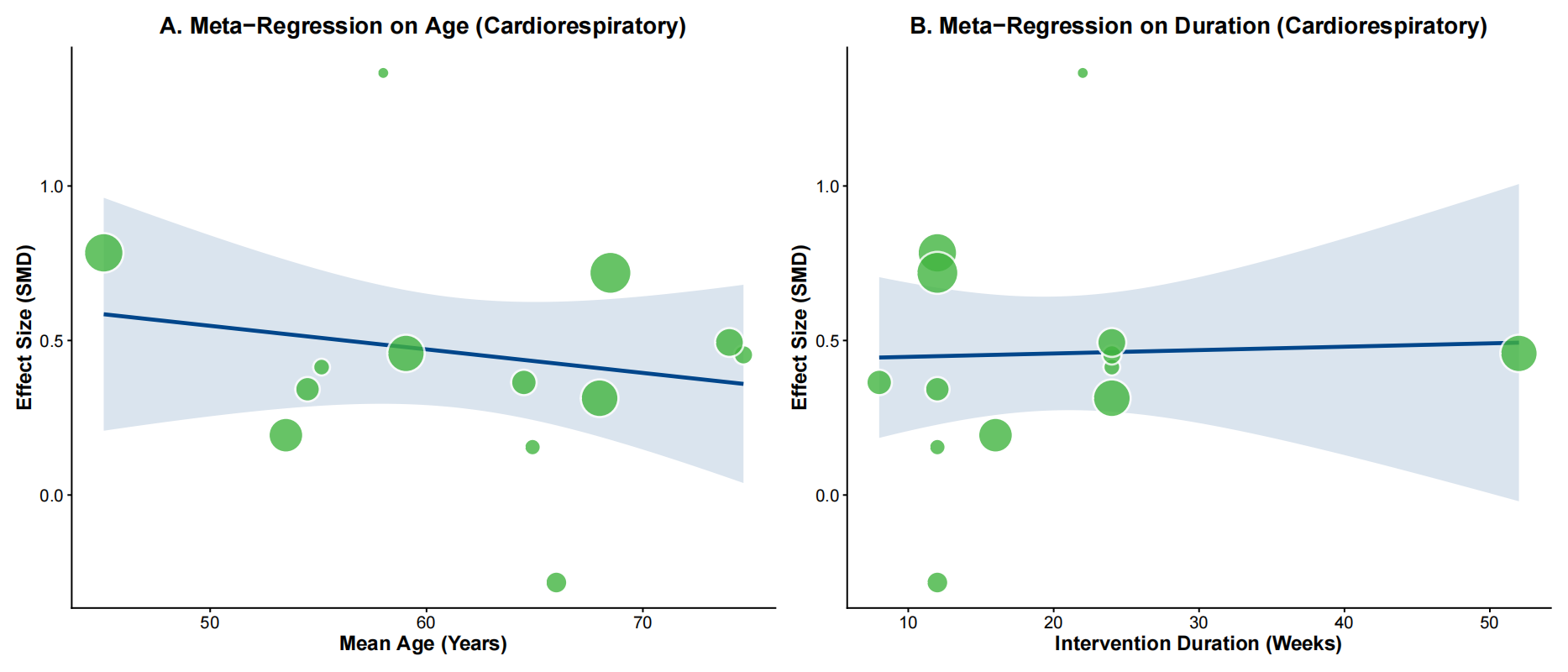

Supplement: Supplementary file 1 [file Presentation_1.zip › Supplementary Material/Figure A5. Meta-regression bubble plots of continuous covariates (A mean patient age; B intervention duration) moderating the effect of exercise interventions on cardiorespiratory fitness..tif]

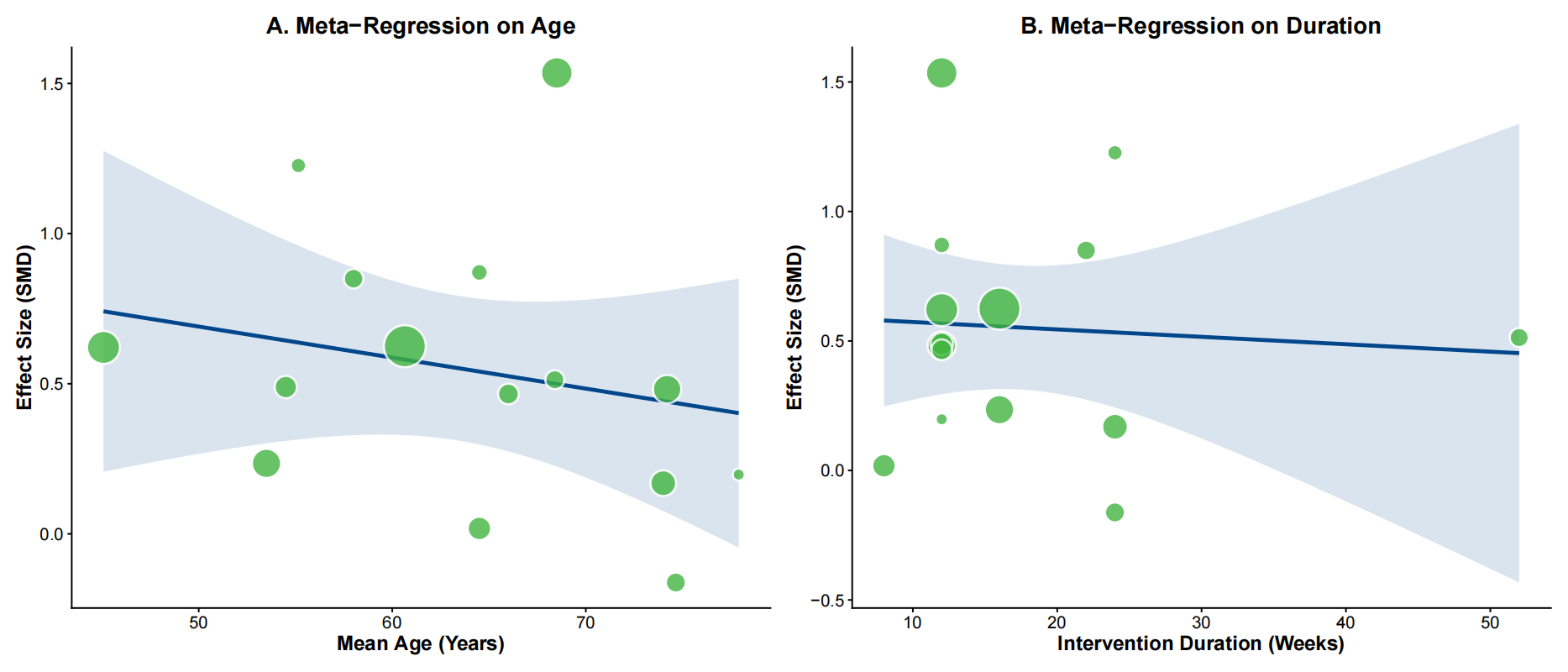

Supplement: Supplementary file 1 [file Presentation_1.zip › Supplementary Material/Figure A6. Meta-regression bubble plots of continuous covariates (A mean patient age; B intervention duration) moderating the effect of exercise interventions on lower limb function..tif]

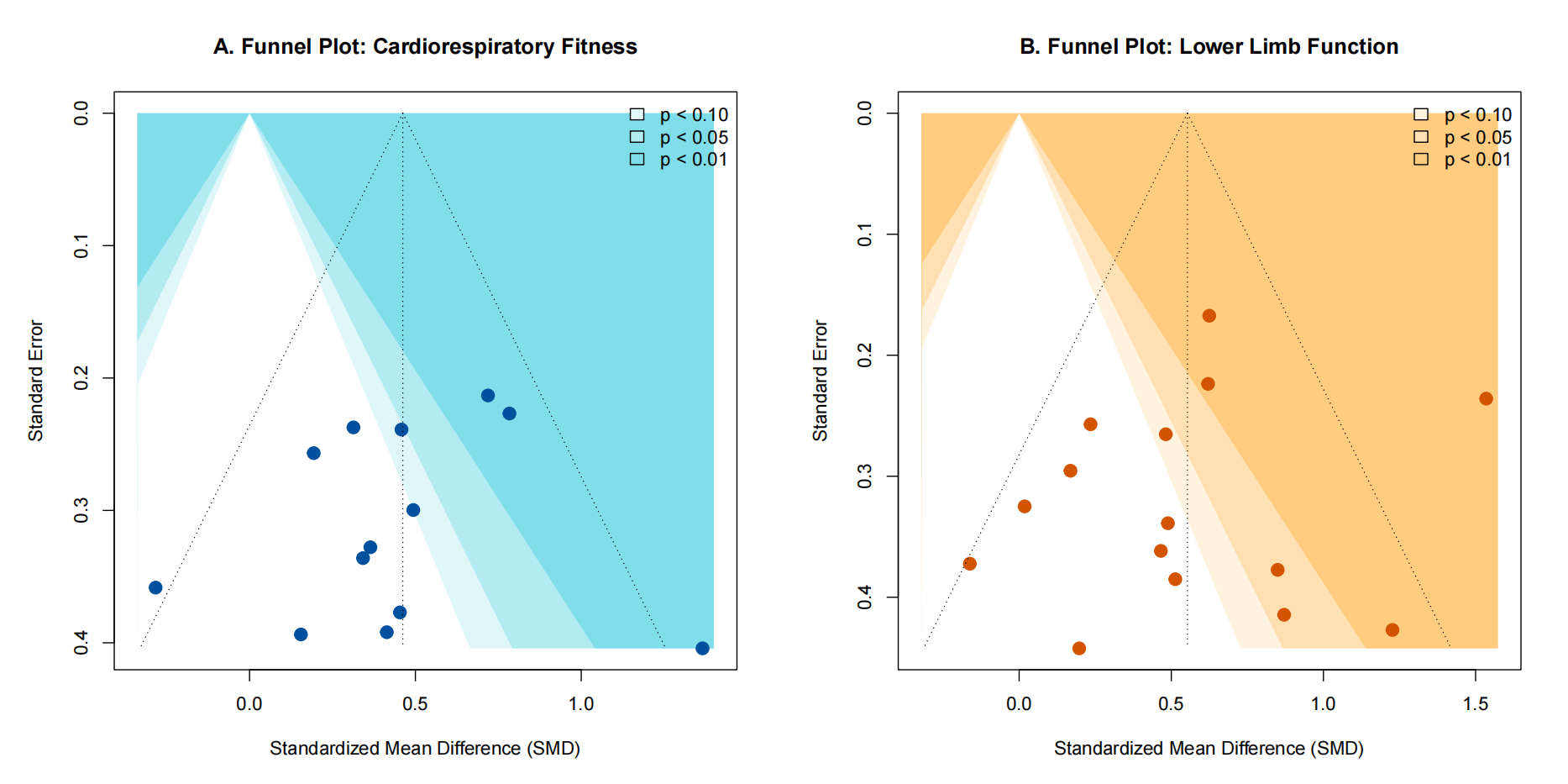

Supplement: Supplementary file 1 [file Presentation_1.zip › Supplementary Material/Figure A7. Funnel plots for the visual assessment of publication bias across the included trials. (A Funnel plot for cardiorespiratory fitness outcomes; B Funnel plot for lower limb function outcomes)..tif]
